# Workplace Violence in a Large Urban Emergency Department

**DOI:** 10.1001/jamanetworkopen.2024.43160

**Published:** 2024-11-05

**Authors:** Marla C. Doehring, Megan Palmer, Ashley Satorius, Tabitha Vaughn, Bruck Mulat, Andrew Beckman, Kyra Reed, Theresa Spech dos Santos, Benton R. Hunter

**Affiliations:** 1 Department of Emergency Medicine, Indiana University School of Medicine, Indianapolis; 2 Michael and Susan Smith Emergency Department, Sidney and Lois Eskenazi Hospital, Indianapolis, Indiana; 3 Faculty Affairs and Professional Development, Indiana University School of Medicine, Indianapolis

## Abstract

**Importance:**

Workplace violence (WPV) against health care workers (HCWs) is common and likely underreported. Reliable data on the incidence of WPV and its impact on victims are lacking.

**Objective:**

To prospectively define the frequency of WPV against HCWs in the emergency department (ED), examine whether HCW demographics are associated with increased risk, and explore the impact of these events on HCWs.

**Design, Setting, and Participants:**

This cross-sectional study was conducted over 2 months in 2023 (August 28 to October 22, 2023) in the ED of a large, urban, academic safety net hospital in the US. Participants included ED physicians, nurses, and other HCWs, who were asked to complete a brief so-called shift sheet for every ED shift worked during the study period.

**Exposure:**

WPV as recorded on shift sheets.

**Main Outcomes and Measures:**

The primary outcome was the number of events per shift. Events were coded for severity (types 1-5) and gender- or race and ethnicity–related bias. Shift sheets asked for the participant’s demographics and whether they experienced verbal or physical abuse during the shift. If so, they were asked to provide a description; rate the impact the event had on them; and indicate whether they felt the event was sexist, racist, or otherwise biased. Perceived impact was recorded, and demographic characteristics associated with the likelihood of experiencing WPV were explored using multivariable logistic regression analysis.

**Results:**

Among 72 HCWs who participated in the study, 52 were female (72%). A total of 575 shift sheets were returned of an estimated 1250 possible (46%), with 155 events, including 77 type 1 events (50%; shouting, yelling, or insults), 29 type 2 events (19%; threats of physical or sexual violence, death threats, or use of slurs), and 39 type 3 events (25%; physical violence); there was a mean (SD) of 3.7 (1.9) shifts per 1 event. No type 4 or 5 events, which involve physical violence causing grievous injuries requiring medical attention and, in the case of type 5 events, permanent disability or death, were recorded. Ten events could not be coded. Sexist or racist bias occurred in 38 events (25%) and 11 events (7%), respectively. Participants reported how the event impacted them in 133 events. Of those, moderate or severe impact was reported in 32 (24%) and mild to no effect in 101 (76%). There was no association between self-reported impact and coded severity of events. In a multivariable logistic regression analysis, a higher likelihood of experiencing WPV on any given shift was independently associated with being in the nursing role (odds ratio, 3.1; 95% CI, 1.9-5.0) and being age 40 years or younger (odds ratio, 2.0; 95% CI, 1.2-3.5).

**Conclusions and Relevance:**

In this cross-sectional study of HCWs in the ED, participants experienced WPV once every 3.7 shifts. The nursing role and younger age were associated with increased risk. These results highlight an urgent need to identify interventions to support and protect HCWs.

## Key Points

**Question:**

How frequently do health care workers (HCWs) in the emergency department (ED) experience workplace violence (WPV), and are there HCW demographics associated with increased odds of experiencing WPV?

**Findings:**

In this cross-sectional study of 72 HCWs in a large urban ED, HCWs experienced WPV once every 3.7 shifts, 25% of which involved physical violence. The odds of experiencing WPV were independently associated with the nursing role and younger age, and participants reported being affected moderately or severely in 24% of events.

**Meaning:**

Results of this study suggest that there is an urgent need to identify interventions to support and protect HCWs, especially those at highest risk.

## Teaser

This cross-sectional study examines how frequently health care workers in the emergency department experience workplace violence and whether their demographic characteristics may be associated with increased odds of experiencing it.

## Introduction

Workplace violence (WPV) is defined by Occupational Safety and Health Administration as any act or threat of physical violence, harassment, intimidation, or other threatening disruptive behavior that occurs at work.^1^ WPV against health care workers (HCWs) is common and likely underreported, partly because estimates of incidence mostly rely on victims’ recall in retrospective surveys.^2,3^ The resulting impact on victims of verbal and physical workplace violence (WPV) is even less well studied. Among HCWs, nurses and female individuals more often experience WPV and may be more negatively impacted by it.^4,5,6,7,8^ Sexist and racist WPV is not well studied but may be especially distressing to those who experience it.^9,10,11^ The consequences of WPV on such individuals can include acute stress, reduced job satisfaction, fear, lost productivity, and ultimately leaving the health care field.^6,8,12^ We sought to prospectively examine the frequency of verbal and physical WPV perpetrated by patients or patient surrogates against HCWs in the emergency department (ED), determine whether HCW demographics are associated with increased risk, and explore the impact of these events on HCWs.

## Methods

This was a 2-month (August 28 to October 22, 2023) cross-sectional study in the ED of a large, urban, academic safety net hospital in Indianapolis, Indiana. It was approved by the Indiana University institutional review board and follows the American Association for Public Opinion Research (AAPOR) reporting guideline for performing and reporting survey research.^13^ HCWs, including emergency medicine faculty, residents, nurses, and other patient-facing staff were recruited to participate in the study using emails and departmental newsletters. Volunteer participants gave written consent via email and were given instructions for completing brief data collection forms, or “shift sheets,” which included additional language regarding informed consent. HCWs were asked to complete a shift sheet (see the eAppendix in Supplement 1) at the end of every ED shift worked during the study period. The sheet asked for the participant’s self-identified demographics, which included their role (eg, nurse or resident), gender (open ended), race and ethnicity (open ended), age by decade (≤30, 31-40, 41-50, and ≥51 years), and whether they experienced verbal or physical abuse during the shift. If no, there were no further questions. If yes, they were asked to provide a brief description; rate the impact the event had on them (none, minimal, moderate, or major); list the location of the incident (eg, the lobby, low acuity, and high acuity); explain whether they felt physically threatened; explain whether they felt there was a racist, sexist, or otherwise biased aspect; and describe whether law enforcement or security was stationed in the patient’s care area prior to the incident. No definitions were provided or mandated for these descriptions, allowing HCWs to report their own perceptions. No mandates were placed on the length or details of the description, to maximize willingness to participate. The form allowed reporting of more than 1 event per shift if necessary.

After the study was completed, each event description was assessed by 2 authors (M.P. and B.R.H.) and coded into different categories (types 1-5) of severity using a previously described instrument.^9,14^ Type 1 events involve shouting, yelling, and insults. Type 2 events are more severe forms of verbal abuse including threats of physical or sexual violence, death threats, or use of slurs. Type 3 events involve physical violence such as kicking, punching, biting, or spitting. Types 4 and 5 events include physical violence causing grievous injuries requiring medical attention and, in the case of type 5 events, permanent disability or death. The 2 authors coded each event blinded to each other’s coding. Discrepancies were resolved through discussion.

### Statistical Analyses

The primary outcome was the frequency of events, calculated and reported as total number of events/total number of shift sheets received. Unadjusted results are reported both for the overall cohort and stratified by different HCW demographics, namely self-identified gender; race (White vs other, as there were a small number of sheets returned by HCWs who reported race or ethnicity other than White; role (nurse, nonphysician practitioner [NPP; equivalent to advanced practice provider, or “APP,” on the shift sheet]), tech, resident physician, faculty physician, and other patient-facing role) and age (≤30 years, 31-40 years, 41-50 years, and ≥51 years). For reporting, tech and other patient-facing roles were combined into 1 category (other). We also used participants’ published work schedules to estimate the number of total shifts worked by the participants during the study period to determine the percentage of shift sheets returned compared to the total number of shifts likely worked.

To determine the independent association between HCW demographics and the likelihood of experiencing a WPV event, we performed a multivariable logistic regression analysis with WPV event as the outcome variable and gender, race and ethnicity, role, and age as the variables. For the multivariable logistic regression analysis, shift sheets with missing demographic data were excluded. The demographic variables were dichotomized. Although gender was asked as an open question, the only answers given were male or female, so only those genders were included; as noted, race and ethnicity were dichotomized into White and other. Nurses represented approximately 50% of the shift sheets returned and reported high rates of WPV, so the category for role was dichotomized into nursing and other role. Different age cutoffs were tested, and the best fit for the model was with age 40 years or younger or 41 years or older, which was used for the final model. Associations of demographic variables with the outcome in the multivariable logistic regression analysis are reported as odds ratios (ORs) with 95% CIs; a 95% CI that does not include 1.0 is considered to be statistically significant. A Hosmer-Lemeshow test was run to determine goodness of fit for the model. The 2-way interactions that were tested in the model-building stage were role × gender, role × age, role × race, gender × age, gender × race, and age × race.

For classification of severity of events 1 through 5, agreement was measured between the 2 authors’ classifications and is reported as κ. Last, we determined whether severity of the event, as coded, was associated with the degree of self-reported impact on the HCW, calculated with impact as a binary variable of either none or minimal vs moderate or major. We calculated the proportion of shift sheets for each type of event in which the HCW reported an impact of moderate or major and compared rates between the types of events using a 2-tailed *t* test with *α* *=* .05 as the level of significance. Analyses were performed using Stata/SE, version 18.0 (StataCorp LLC).

## Results

There were 72 HCWs who participated, including 21 faculty physicians (29%), 11 residents (15%), 30 nurses (42%), 3 nonphysician practitioners (4%), and 7 other hospital employees (10%). Participants included 52 female (72%) and 20 male (28%) HCWs, of whom 66 (92%) were White and 3 (4%) were categorized as other. Over the 2-month study period, 575 shift sheets were returned, which was 46% of the estimated 1250 shifts worked by participants based on published schedules. There were 155 events reported in the 575 shift sheets, for a mean (SD) of 3.7 (1.9) shifts per 1 event. Most of the events (n = 77 [50%]) were coded as type 1 verbal abuse, but 39 (25%) involved physical violence or assault (type 3 events). There were no instances of type 4 or 5 incidents. Ten events (6%) lacked enough description to determine severity or whether they would clearly meet the definition of WPV; 29 (19%) involved more severe forms of verbal abuses (type 2 events). Agreement for coding event severity was almost perfect (κ = 0.84). Some examples of event descriptions are provided in the Box. Sexist and racist bias was frequent, occurring in 38 events (25%) and 11 events (7%), respectively. Of the 133 events that had impact reported, participants felt the WPV they had experienced affected them moderately or severely in 32 events (24%) and had mild to no effect in 101 (76%) (Figure).

Box. **Representative Comments by Incident Type**

**Type 1 (Shouting, Yelling, and Insults)**

1. Patient repeatedly yelling in room. Called nurse “mother f----r” and “fat b---h” while the nurse was attempting to assist the patient.
2. Patient called staff a “Stupid white ass b---h. I said I am in pain; Tylenol is not going to do s--t.” then yelled “Stupid little b---h don’t come back in my room until you have some real pain meds.”
3. Angry and hostile toward staff, refused care then loudly screamed at staff for not doing anything, cussed at staff multiple times.

**Type 2 (Threats of Physical or Sexual Violence, Death Threats, Use of Slurs)**

1. Patient told staff “I’ll f-----g kill you.”
2. Pt called me a b---h said we all need bedside manner classes; said I was lucky he didn’t beat my ass.
3. As I was placing the line the patient continued to make comments like “I bet you like getting poked with big things. I got a big thing to poke you with.”

**Type 3 (Physical Violence Such as Kicking, Punching, Biting, Spitting)**

1. Patient agitated, lunged toward provider while attempting to rip out IV.
2. Patient physically assaulted CIU employee and broke computers.
3. The patient twice tried to bite staff, hit the nurse on rib cage, and pinched skin on her arm.

**Sexist**

1. Patient was asking for restraints to be removed because “I can get my hands on your body. You have a nice ass. I bet you taste good baby.”
2. Patient repeatedly screamed “F--k you…” calling staff…, “4 eyed nerd,” “bimbo b---h,” and “gutter slur.” Patient physically aggressive and physically fighting security.
3. “Little young ass m-----------s don’t know s--t. Rude little b-----s need taught a lesson, like you! Women don’t know s--t.”

**Racist**

1. Pt intoxicated and using racial slurs like n----r and being aggressive toward staff.
2. Visitor rolling eyes at Hispanic RN and then walked over to registration and asked if they spoke English and continued to engage in conversation with them and disregarded efforts made to assist visitor with locating patient by RN, who was speaking in English.
3. Patient states, “I have an emergency.” Patient then states “I matter too. I am American” (nurse was registering a different patient who was Latinx). The nurse asks patient to stop interrupting. Patient states “You are rude…you shouldn’t be working up here.”

CIU indicates crisis intervention unit; IV, intravenous line; RN, registered nurse.

**Figure.**
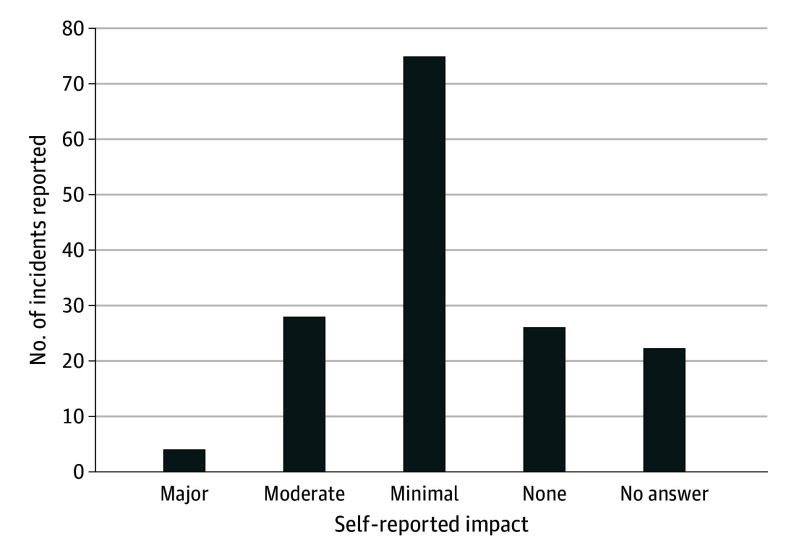
Self-Reported Impact of Workplace Violence Incidents on Health Care Workers

The number of shift sheets returned and unadjusted frequencies of WPV stratified by HCW demographics are displayed in Table 1. Of the 575 returned shift sheets, 465 (81%), representing 120 WPV events had complete HCW demographic data and could be included in the multivariable logistic regression analysis. More shift sheets were turned in for self-identified White (421 sheets [73%] for White vs 53 [9%] for other HCWs) and female (372 sheets [65%] for female vs 128 [22%] for male) HCWs. Nurses represented 46% of shift sheets returned (262 sheets). Numerically, WPV was more common among White, female, nurse, and younger HCWs (Table 1). In the multivariable logistic regression analysis, age 40 years or younger (OR, 2.0; 95% CI, 1.2-3.5) and nursing role (OR, 3.1; 95% CI, 1.9-5.0) were associated with increased odds of experiencing WPV. Race and ethnicity (OR, 0.4; 95% CI, 0.2-1.0 for those categorized as other) and gender (OR, 0.9; 95% CI, 0.5-1.8) were not associated with WPV frequency. The Hosmer-Lemeshow test showed that the model was a good fit (χ^2^ statistic of 1.80; *df* = 4; *P =* .77). Including interaction terms resulted in worse fit of the model, and no multicollinearity issues were observed.

**Table 1.** Summary of Incidents by HCW Demographic Characteristics

Characteristic	No. of incidents/No. of shifts (%)	No. of shifts per 1 incident, mean (SD)^a^
Race and ethnicity		
White	115/421 (27)	3.7 (1.9)
Other^b^	7/53 (13)	7.6 (2.8)
No response	33/101 (33)	3.1 (1.7)
Gender^c^		
Female	117/372 (31)	3.2 (1.8)
Male	21/128 (16)	6.1 (2.5)
No response	17/75 (23)	4.4 (2.1)
Role		
Faculty physician	24/165 (15)	6.9 (2.6)
Resident	11/43 (26)	3.9 (2.0)
Nurse	110/262 (42)	2.4 (1.5)
Nonphysician practitioner^d^	3/31 (10)	10.3 (3.2)
Other	4/66 (6)	16.5 (4.1)
No role specified	3/8 (38)	2.7 (1.6)
Age, y		
≤30	80/183 (44)	2.3 (1.5)
31-40	41/179 (23)	4.4 (2.1)
41-50	15/106 (14)	7.1 (2.7)
≥51	18/99 (18)	5.5 (2.3)
No age specified	1/8 (13)	8.0 (2.8)

^a^ Mean and SD calculated assuming Poisson distribution.

^b^ Participants of other races and/or ethnicities were collapsed into this group because of small numbers.

^c^ Participants could have given other answers than male or female, but none did.

^d^ Called “APP” (advanced practice provider) on the shift sheet.

Table 2 shows the self-reported impact on HCWs stratified by type of WPV event for the 129 events that could be coded for severity and had impact reported by the participant. Impact was rated as moderate or major in 23% (13 moderate and 2 major), 31% (9 moderate, no major), and 24% (7 moderate and 2 major) of type 1, 2, and 3 events, respectively. None of these differences were statistically different from each other (*P* value for type 1 vs type 2 = .26; type 2 vs type 3 = .47; type 1 vs type 3 = .85).

**Table 2.** Impact of Workplace Violence on Participants by Severity Type^a^

Impact	Events, No. (%) (N = 129)^b^		
	Type 1 (n = 66)	Type 2 (n = 26)	Type 3 (n = 37)
None	12 (18)	5 (19)	9 (24)
Minimal	39 (59)	12 (46)	19 (51)
Moderate	13 (20)	9 (31)	7 (19)
Major	2 (3)	0 (0)	2 (5)

^a^ P value for type 1 vs type 2 = .26; type 2 vs type 3 = .47; type 1 vs type 3 = .85.

^b^ Of 155 total events, there were 26 events that were either missing impact data or were unable to be coded for severity from the description provided.

## Discussion

Consistent with previous studies of WPV against HCWs, this prospective cross-sectional study found that nurses were more likely to experience WPV compared to physicians or HCWs in other roles.^4,6^ In our study, HCWs in the ED often experienced WPV once every 3.7 shifts worked. Assuming that a typical full time HCW works 15 shifts per month, this would represent experiencing WPV approximately 4 times a month or once per week. Of these incidents, 25% involved physical violence, similar to previous studies of WPV against HCWs.^9,10,15^ There are few prospective studies of WPV in EDs in the US. A 2013 study reported a lower rate of violent events per worker, approximately 1 event every 2 months.^6^ This could be due to different definitions of WPV or lack of recall since surveys were sent only monthly rather than requested at the end of every shift. Additionally, there is evidence that WPV in health care may be increasing, especially since the COVID-19 pandemic.^16,17^

Although less well-studied, incidents involving younger HCWs were more common which is also consistent with earlier work.^8^ Importantly, almost one-quarter of participants in our study disclosed that the event had a moderate or major impact on their personal wellness. This level of impact is a significant problem that can lead to acute stress, lost productivity, burnout, and ultimately leaving the health care field.^6,7,8^

Sexist or racist bias was reported in approximately one-third of incidents in this study. Biased WPV is not well studied but is especially disturbing as health care organizations work to increase the diversity of their workforces. Racial bias in this study was similarly or less frequent compared with previous studies, while sexist bias was more common.^9,10,11,15^ Future studies could investigate perpetrator characteristics to help identify those at risk of becoming violent and, even more importantly, how to prevent WPV.

The evidence regarding the effectiveness of interventions to reduce and prevent WPV in the ED is limited. Several small studies have evaluated a variety of educational programs for HCWs and found them somewhat helpful in decreasing the frequency of violent incidents or improving how prepared staff felt in their ability to deal with WPV events.^18,19,20^ A multipronged approach to addressing WPV is crucial. Educational programs, environmental factors (eg, controlled access, signage, and furniture arrangement) and organizational strategies (eg, hospital policies, adequate staffing, and governmental policies) may be helpful and require future study to evaluate their effectiveness. Future work to mitigate and prevent workplace violence in health care will require support from multidisciplinary teams including HCWs, administrators, legal experts, and policy makers. In addition, biased WPV against HCWs and the personal impact of WPV on HCWs should be specifically studied as health care organizations work to retain their highly skilled workforce and support a more diverse and inclusive health care team.

### Limitations

There are several limitations to this study. Not all invited HCWs decided to participate in the study, and those who did not may have been more or less prone to experience or report WPV. We had no way of capturing incidents or shifts with no incidents unless shift sheets were returned. We estimated that shift sheets were turned in on approximately half of shifts worked by participants. It is possible that this represents selection bias whereby shifts without an event were less likely to be reported. Even if all shifts worked without a returned shift sheet included zero incidents, the frequency of WPV would be approximately once every 7.5 shifts, still an alarming frequency that would correlate with approximately 2 events per month for a full time ED HCW, still higher than in previous studies.^6,10^ Another limitation is that some forms had incomplete data, such as demographics, which was expected, given the open-ended nature of the reporting forms. To maximize response rates, we did not require completion of all fields. Unfortunately, this resulted in 110 sheets being excluded from our multivariable logistic regression analysis. The frequency of events in excluded shift sheets was similar to those with complete demographic data. For ease of use and to maximize participation, we asked participants to complete 1 data collection sheet per shift. Data regarding number of hours per shift and the time of the day of each shift were not collected. However, the HCWs who participated in this study included those who worked rotating shifts throughout the week and at all times of the day, in addition to those who worked only day shifts or only night shifts. The dataset includes repeated observations of HCWs, which may violate the independence assumption of the logistic regression model. Ideally, mixed-effects models could be used to account for these repeated measures, but by design, individual shift sheets could not be traced to any individual HCW, so we could not account for repeated measures.

Coding of events was not entirely objective, although we followed a previously described rubric,^9,14^ and 2 study personnel independently coded all events with near-perfect agreement. Outside of our coding process, there was no definition provided to participants of what constitutes WPV. Using observers with a more defined definition of WPV might have increased precision in terms of what constitutes WPV. However, perhaps even more meaningful than a standardized definition of WPV, each event was seen by the HCW participant as impactful enough to report and describe as such. The small number of non-White HCWs and lack of gender diverse HCWs in this study may also limit generalizabilty to more diverse populations of HCWs. The study took place in a large urban ED with 24/7 security presence, and the results may not be widely generalizable to other settings.

## Conclusions

In this cross-sectional study of HCWs in the ED, participants reported experiencing WPV with alarming frequency. This may contribute to high rates of attrition and burnout of a skilled and highly trained workforce. Gender- and/or race and ethnicity–biased WPV was not uncommon. It is imperative that future studies identify strategies to protect our HCWs, especially those who may be at highest risk, including younger HCWs and nurses.

## Notes

Supplement 1. **eAppendix.** Data Collection Sheet

Supplement 2.
Data Sharing Statement
