# Supplementary material for: Workplace Violence in a Large Urban Emergency Department
Source: JAMA Netw Open. 2024 Nov 5;7(11):e2443160. doi: 10.1001/jamanetworkopen.2024.43160 (PMC11539014; doi:10.1001/jamanetworkopen.2024.43160)
Supplement: Supplement 1. — eAppendix. Data Collection Sheet [file jamanetwopen-e2443160-s001.pdf]

Supplemental Online Content

Doehring MC, Palmer M, Satorius A, et al. Workplace violence in a large, urban emergency department. *JAMA Netw Open*. 2024;7(11):e2443160. doi:10.1001/jamanetworkopen.2024.43160

eAppendix. Data Collection Sheet

This supplemental material has been provided by the authors to give readers additional information about their work.

© 2024 Doehring MC et al. *Jama Network Open.*

eAppendix. Data Collection Sheet

This form is only for participants who have agreed to enroll in this study and commit to fill out a form for each of their shifts during the study period. If you have not enrolled in this study and want to enroll, please email XXXXXX

Data Collection Sheet: Frequency of Workplace Violence in the ED

- You are being asked to participate in a research study which aims to define the frequency with which healthcare workers (HCWs) experience workplace violence during shifts in the ED.
- There are no direct benefits to participants. The information gathered from the study may inform future work and future protocols at Eskenazi Hospital, so there is some potential that after the study there will be changes made to further protection for HCWs, but this would be on a system level, and there are no anticipated benefits to participants on an individual basis.
- We also anticipate no risks to participants. There is a small risk that the participant could be identified as the victim of an abusive event. If the participant is concerned about this, they are allowed to not fill out demographic questions and not provide a patient sticker, in which case identification of the participant would be realistically near zero.
- Participation in this study is strictly voluntary. Whether or not you choose to participate in this study will have no effect on your employment and/or educational status.
- Contact XXXXXXXX with any questions about this study.
- Please complete one form per shift regardless of whether you experienced abuse. All questions are optional. At the end of your shift, please return this sheet to the area where yellow patient safety forms are collected.

Your Role: Nurse APP Tech Resident physician Faculty physician Other patient-facing role

Your Gender: *Your Race:*

Your Age: ≤30 31-40 41-50 ≥51

Did you experience verbal or physical abuse during this shift? YES NO

IF YES, PLEASE DESCRIBE BELOW. IF NO, PLEASE STOP AND TURN IN THIS FORM. THANK YOU for your participation!

Incident #1:

Description of abusive incident: ____________________

Patient Name/MRN
(If possible)

____

____

____

____

____

Location: Lobby Swat Intake Low Acuity High Acuity Shock Rm Holding Other:__________

Did you feel physically threatened? YES NO

Did you feel abuse was biased in nature? YES NO If yes, please circle which type of bias and describe the biased abuse. SEXIST RACIST OTHER BIAS (_________)

____

____

____

© 2024 Doehring MC et al. *Jama Network Open.*

Was law enforcement or security stationed in the patient's care area prior to the incident today (i.e. – was the patient in LA South or Shock rooms with an officer stationed nearby prior to the incident?): YES NO NOT SURE

How much did this abusive incident negatively impact how you felt for the rest of your shift?
No impact Minimal impact Moderate impact Major impact

Incident #2:
Your description of abusive incident:

Patient Name/MRN<br>(If possible)

____

____

____

____

____

____

Location: Lobby Swat Intake Low Acuity High Acuity Shock Rm Holding Other:__________
Did you feel physically threatened? YES NO
Did you feel abuse was biased in nature? YES NO If yes, please circle which type of bias and describe the biased abuse. SEXIST RACIST OTHER BIAS (_________)

____

____

____

Was law enforcement or security stationed in the patient's care area prior to the incident today (i.e. – was the patient in LA South or Shock rooms with an officer stationed nearby prior to the incident?): YES NO NOT SURE

How much did this abusive incident negatively impact how you felt for the rest of your shift?
No impact Minimal impact Moderate impact Major impact

*** If you had 3 or more abusive incidents during this shift, please staple additional forms to this one with details for each incident.*

© 2024 Doehring MC et al. *Jama Network Open.*
