# Supplementary material for: Workplace Violence in a Large Urban Emergency Department
Source: JAMA Netw Open. 2024 Nov 5;7(11):e2443160. doi: 10.1001/jamanetworkopen.2024.43160 (PMC11539014; doi:10.1001/jamanetworkopen.2024.43160)
Supplement: Supplement 2. — Data Sharing Statement [file jamanetwopen-e2443160-s002.pdf]

Data Sharing Statement

Doehring. Workplace Violence in a Large Urban Emergency Department. *JAMA Netw Open*. Published November 05, 2024. doi:10.1001/jamanetworkopen.2024.43160

Data
Data available: No
